# Emerging Roles of miRNA, lncRNA, circRNA, and Their Cross-Talk in Pituitary Adenoma

**DOI:** 10.3390/cells11182920

**Published:** 2022-09-19

**Authors:** Wentao Wu, Lei Cao, Yanfei Jia, Youchao Xiao, Xu Zhang, Songbai Gui

**Affiliations:** 1 Department of Neurosurgery, Beijing Tiantan Hospital, Capital Medical University, 119 South Forth West Ring, Beijing 100070, China; 2 Department of Oncology, First Affiliated Hospital of Anhui Medical University, 218 Jixi Road, Hefei 230032, China

**Keywords:** ceRNA, biomarker, non-coding RNA, pituitary adenomas, pathogenesis

## Abstract

Pituitary adenoma (PA) is a common intracranial tumor without specific biomarkers for diagnosis and treatment. Non-coding RNAs (ncRNAs), including microRNAs (miRNA), long non-coding RNA (lncRNA), and circular RNA (circRNA), regulate a variety of cellular processes, such as cell proliferation, differentiation, and apoptosis. Increasing studies have shown that the dysregulation of ncRNAs, especially the cross-talk between lncRNA/circRNA and miRNA, is related to the pathogenesis, diagnosis, and prognosis of PA. Therefore, ncRNAs can be considered as promising biomarkers for PA. In this review, we summarize the roles of ncRNAs from different specimens (i.e., tissues, biofluids, cells, and exosomes) in multiple subtypes of PA and highlight important advances in understanding the contribution of the cross-talk between ncRNAs (e.g., competing endogenous RNAs) to PA disease.

## 1. Introduction

Pituitary adenoma (PA) is the third most common intracranial tumor, accounting for 10 to 15% of all intracranial tumors [1]. The prevalence of PA is 1:10,000 [2]. In addition, it has been shown that the average prevalence of PA is 14.4% in autopsies and 22.5% in radiological series [2]. PAs are usually benign lesions, but some patients have aggressive characteristics, including local invasiveness, rapid growth, and poor responses to conventional treatments [3]. Consequently, they are divided into functional PAs (FPAs) and non-functional PAs (NFPAs). FPAs include growth hormone (GH)-, prolactin (PRL)-, adrenocorticotropic hormone (ACTH)-, thyroid-stimulating hormone (TSH)-, and follicle-stimulating hormone/luteinizing hormone (FSH/LH)-secreting adenomas [4,5]. Some proteins are involved in the pathogenesis of PA subtypes. Patients with either familial or sporadic PA often have aryl hydrocarbon receptor-interacting protein mutations, and guanine nucleotide-binding protein G (s) subunit alpha and G-protein-coupled receptor 101 mutations contribute to excess growth hormone [6]. In addition, abnormal expression levels of the deubiquitinases BRAF, USP8, and USP48 are related to the production of adrenocorticotropic hormone [6]. However, many patients with PA lack early biomarkers due to endocrine diseases and nerve compression [1]. In addition, patients with PA are difficult to identify in the existing medical diagnosis, and many adverse reactions occur during the treatment of patients with PA [7]. Therefore, it is of great clinical significance to further investigate the pathogenesis of PA and find novel molecular targets for the diagnosis and treatment of PA. In recent years, epigenetics, especially non-coding RNAs, has been demonstrated to play an important role in the pathogenesis of PA.

About 75% of the human genome can be transcribed, but only 1.5% of the transcribed sequences code for proteins; i.e., over 98% of non-coding sequences do not have the biological function of coding for proteins (named non-coding RNAs, ncRNAs) [8,9,10,11]. Although ncRNAs cannot encode proteins, they regulate the protein expression processes, including transcription and translation. Furthermore, increasing studies have shown that ncRNAs are involved in the multilevel regulation of gene expression and have a key role in the central dogma of molecular biology [12,13]. Thus, ncRNAs regulate cell differentiation, development, and cellular fate [13]. The most common ncRNAs include microRNA (miRNA), long non-coding RNA (lncRNA), and circular RNA (circRNA), which are all actively involved in the regulation of tumor-suppressor genes and oncogenes [14,15,16]. Numerous studies have suggested that the abnormal expression of ncRNAs is related to the occurrence and progression of PA, and exosome-derived ncRNAs have enriched our understanding of the pathogenesis of PA. Consequently, ncRNAs, especially exosome-derived ncRNAs, may have a key role in the pathogenesis of PA and may become novel diagnostic and therapeutic biomarkers.

In this article, we summarize the biological significance of ncRNAs, exosome-derived ncRNAs, and their target genes in different PA subtypes (e.g., NFPA, IPA, GHPA, etc.). In addition, we also discuss the potentiality of ncRNAs as noninvasive biomarkers for the diagnosis and prognosis of PA.

## 2. miRNA and PA

The miRNA, a small, endogenous, single-stranded, non-coding RNA with a length of 19 to 25 nucleotides that inhibits post-transcriptional protein synthesis via binding to the 3′-untranslated region (UTR) of target messenger RNAs (mRNAs), plays an important role in a variety of essential and biophysiological processes including cell proliferation, differentiation, and apoptosis. Accumulating studies have shown the dysfunction of miRNAs in human biological fluids and in cell-free environments, suggesting that these miRNAs can function as oncogenes and tumor suppressors. These important findings may contribute to provide novel diagnostic and prognostic biomarkers for PA. Table 1 summarizes miRNAs differentially expressed (DE) in PA and its subtypes.

**Table 1:** Dysregulation of miRNA in different PA types.

Author (Ref.)	Type of PA	Sample	miRNA	Expression	Target Gene	Biological Function
Mossakowska [17]	CD	Tissues, AtT-20/D16v-F2 cells	hsa-miR-124-3p and hsa-miR-135-5p	Down	NR3C1, NR3C2	Regulation of glucocorticoid receptor
Zhang [18]	NFPA	Serum	miRNA-26b, miRNA-138, miRNA-206, and let-7e	Down	NA	Tumor-suppressor gene
Chen [19]	PA	Tissues, GH3 cells	miR-424-3p	Down	JAG1	Cell proliferation and EMT
Zhao [20]	IPA	Exosomes, tissues, and GH4C1, MMP, and GH3 cells	miR-149-5p and miR-99a-3p	Down	NOVA1, DTL, RAB27B	The expression of EMT- and ECM-related markers and tumor-related genes
Wei [21]	PA	Tissues, GH3 cells	miR-137	Down	EGFR	Tumor suppressor, cell proliferation
Wu [22]	IPA	Tissues	miR-193a-3p	Down		Higher risk of postoperative residual and recurrence
Wang [23]	PA	GT1.1 and AtT-20 cells	miR-219a-2-3p	Down	MDM2	Cell proliferation and apoptosis
Németh [24]	GHPA, FSH/LH+ PA, hormone-immunonegative PA	Plasma	miR-143-3p	Down	NA	Distinguishing preoperative plasma samples from normal controls
He [25]	NFPA, GHPA, PRLPA	Tissues	miR-34c-3p, miR-34b-5p, miR-378, miR-338-5p, miR-493-5p, miR-181b-5p, miR-184, and miR-124-3p	PRLPA: miR-34c-3p, miR-34b-5p, miR-378, and miR-338-5p were downregulated; NFPA: miR-493-5p and miR-124-3p were downregulated and miR-181b-5p was upregulated; GHPA: miR-184 was upregulated and miR-124-3p was downregulated	NA	Novel biomarkers
Cai [26]	NFPA	Tissues	miR-370	Down	HMGA2	miR-370 was decreased by CXCL12 treatment and miR-370 inhibited tumor growth and invasiveness
Song [27]	NFPA	Tissues, GH3 cells	miR-137	Down	WIF1	Cell proliferation and invasion
Leone [28]	GHPA, NFPA		miR-23b and miR-130b	Down	HMGA2, CCNA2	Cell proliferation arresting the cells in the G1 and G2 phase of the cell cycle
Gentilin [29]	ACTH-secreting PA	Tissues, AtT-20 cells	miR-26a	Up	PRKCD	Delayed the cell cycle in G1 phase
Trivellin [30]	GHPA, NFPA	Tissues, GH3 cells	miR-107	Up	AIP	Cell proliferation
Butz [31]	NFPA	Tissues	miR-135a, miR-140-5p, miR-582-3p, miR-582-5p, and miR-938	Up	Smad3	Tumor size
Butz [32]	NFPA, GHPA	Tissues	miR-128a, miR-155, and miR-516a-3p	Up	Wee1	Cell proliferation
Qian [33]	PA	Tissues	let-7	Down	HMGA2	Tumor invasion
Bottoni [34]	GHPA, PRLPA	Tissues	miR-15a and miR-16-1	Down		Tumor diameter
Lyu [35]	NFPA	Exosome	hsa-miR-486-5p	Down	NA	Tumor progression
He [36]	PA	Tissues, MMQ and HP75 cells	miR-448	Down	BCL2	Cell proliferation and migration
Xiong [37]	GHPA	Exosome, tissues	hsa-miR-21-5p	Up	PDCD4	Bone formation and trabecula number
Yang [38]	PA	GH4C1 cells	miR-34a	Down	Sox7	Cell proliferation and apoptosis
Fan [39]	GHPA	Tissues, GH3 cells	miR-185	Up	SSTR2	Cell proliferation and apoptosis
Liao [40]	PA	GH3 and MMQ cells	miR-200c	Up	PTEN	Pituitary tumor formation

Ref., reference; NA, not available; EMT, epithelial–mesenchymal transition; ECM, extracellular matrix.

### 2.1. Circulating miRNA and PA

In 2008, circulating miRNA (c-miRNA) was detected in peripheral blood [41,42,43]. Compared to that of cellular RNA, the expression of c-miRNA is very stable in the RNase-rich environments of human biofluids due to its binding to specific proteins (e.g., the Argonaute (Ago 2) protein) [44,45]. In addition, c-miRNAs have been shown to be resistant to harmful conditions, such as high temperature, acidic or alkaline environments, and repeated freeze–thaw cycles [41,46]. Therefore, c-miRNAs are ideal candidates for novel noninvasive blood biomarkers for different types of diseases, and c-miRNAs contribute to diagnosis, prognosis, and postoperative monitoring for PA with aggressive behavior and NFPA.

The RNA sequencing (RNA-seq) of plasma samples showed that there were several DE miRNAs in preoperative and postoperative patients with PA, including 3 DE miRNAs in a GH group, 7 in an FSH/LH group, and 66 in a hormone-immunonegative (HN) group [24]. Subsequently, some DE miRNAs (i.e., miR-143-3p in FSH/LH, and miR-26b-5p, miR-126-5p, and miR-148b-3p in HN) were used for validation by real-time polymerase chain reaction (qRT-PCR) [24]. Circulating miR-143-3p was significantly upregulated in patients with preoperative FSH/LH compared to patients with postoperative FSH/LH+ [24].

Furthermore, the tumor size in patients with postoperative FSH/LH decreased significantly, which may result in a decrease in circulating miR-143-3p [24]. Circulating miR-143-3p exhibited a strong differentiation power, with an area under curve (AUC) value of 0.79 between preoperative and postoperative patients with FSH/LH PAs [24]. The plasma miR-143-3p decreases in patients with FSH/LH+ adenoma, but its application in the evaluation of tumor recurrence needs further investigation [24]. These findings suggest that plasma miR-143-3p decreases may be a potential biomarker for patients with FSH/LH+ adenoma after transsphenoidal surgery. In addition, some studies have also found that miR-143 was significantly downregulated in PA tissues [47,48], and inhibited tumor proliferation by targeting the K-Ras gene [47].

Belaya et al. evaluated whether miRNAs were DE in plasma samples from patients with adrenocorticotropic hormone (ACTH)-dependent Cushing’s syndrome (CS) caused by either ectopic ACTH secretion (EAS) or Cushing’s disease (CD) [49]. In their study, 21 miRNAs were measured [49]; the circulating levels of miR-16-5p, miR-145-5p, and miR-7g-5p were altered; and miR-16-5p had the most distinguished power, with an AUC value of 0.879. Therefore, these results indicate that c-miRNAs are promising biomarkers for distinguishing between CD and EAS.

In addition to biomarkers for diagnosis and prognosis, c-miRNAs were also associated with the survival time for PA. Compared to that in healthy controls, the serum expression of miR-16 was decreased in patients with PA, whereas higher levels of miR-16 were accompanied by longer overall survival (OS) times and disease-free survival (DFS) times [50]. In vitro, miR-16 inhibited the proliferation and angiogenesis of PA via regulating the VEGFR2/p38/NF-κB pathway [50]. Consequently, miR-16 may be a potential target for the treatment of PA.

In conclusion, c-miRNAs have an important role for the diagnosis and prognosis of PA, but also for the treatment of PA. In the future, the research directions of c-miRNAs may lead to the translation of potential candidates with important roles in the development of the disease to their implementation as biomarkers for the diagnosis and prognosis of PA in clinical practice.

### 2.2. miRNA in PA Tissue

Some studies have shown that the abnormal expression of miRNAs in tumor tissues was significantly associated with the levels of miRNAs released into the peripheral circulation [1,7,51], suggesting that the change in miRNAs in PA tissues is implicated in the occurrence and progression of the disease.

In 2005, several DE miRNAs between normal pituitary tissues and PAs were identified [34]. Subsequent studies have shown that miRNAs may be involved in tumorigenesis, invasion, and aggressiveness as oncogenes and as tumor suppressors. Using the HiSeq 2000 sequencing system, several distinctive miRNA expression patterns were identified in three different PAs (i.e., NFPAs, GHPAs, and PRLPAs) [25]. Compared to normal pituitary tissues, significant downregulation of miR-34c-3p, miR-34B-5p, miR-378, and miR-338-5p in PRLPA; downregulation of miR-493-5p and upregulation of miR-181b-5p in NFPA; and significant upregulation of miR-184 in GHPA were observed. In addition, downregulation of miR-124-3p in both NFPA and GHPA was observed [25]. These miRNA signatures may be promising therapeutic biomarkers for different types of PA. A few studies investigated aggressiveness-associated miRNAs in aggressive pituitary tumors; several DE miRNAs between PRLPA and aggressive PA were identified using the GSE46294 miRNA expression profile [52,53]. They suggested that most of the hub genes were modulated by hsa-miR-489 and hsa-miR-520b via the construction of a miRNA–hub gene network; thus, these two miRNAs may provide new targets for the diagnosis and treatment of PRLPA.

In addition to being downregulated in the PAs’ plasma, miR-143 was downregulated in PA tissues, especially in ACTH-secreting pituitary tumors [24,47,48]. Although the expression of miR-143 was not associated with the tumor size and postoperative remission rate [48], miR-143 inhibited cell proliferation and promoted apoptosis by targeting K-Ras [47]. Vicchio et al. found that the downregulation of miR-26b-5p and miR-30a-5p was negatively correlated with ki-67, ‘atypical’ morphological characteristics, and cavernous sinus invasion [54]. These miRNAs can be used as predictors of PA invasion.

In conclusion, exploring the underlying mechanisms between miRNAs and pituitary tumorigenesis may contribute to identifying new potential biomarkers that may be used as an innovative treatment for PA.

### 2.3. miRNA in PA-Associated Cell Lines

PA-associated cell lines including mouse (e.g., AtT-20 and GT1.1 cells) and rat (e.g., GH3 and MMQ cells) lines were used for exploring the underlying mechanisms of PA. The expression of miR-143 was downregulated in the GH3 and MMQ cell lines except in the circulation and tissue [24,47,48]. Subsequent experiments revealed that miR-143 inhibited the two cells’ proliferation and promoted apoptosis by regulating the oncogene K-Ras [47]. Furthermore, a recent study found that the underexpression of miR-146b-5p was related to the tumor size, the overall survival rate, a poor disease-free survival rate, a poor Knosp grade, and a poor Hardy grade [55]. In addition, miRNA-146b-5p negatively regulated GH3 cell proliferation, invasion, and migration, and induced apoptosis by inhibiting the ephrin receptor A7 (EPHA7) gene via regulating the IRAK4/TRAF6/NF-κB signaling pathway [55].

In murine GT1.1 and AtT-20 cells, the expression of miR-219a-2-3p was significantly downregulated [23]. Moreover, the overexpression of miR-219a-2-3p inhibited cell proliferation and promoted apoptosis as well as reducing MDM2 expression by binding to the 3’-UTR of the MDM2 mRNA and promoting p53 expression [23]. Therefore, miR-219a-2-3p modulated cell proliferation and apoptosis by targeting MDM2/p53 in PA, suggesting that miR-219a-2-3p may be a novel therapeutic marker for PA.

In conclusion, in vitro studies provide greater insight into the pathogenesis of the disease, and the combination of in vitro, in vivo, and clinical specimens (including blood and tissue) will greatly promote the research progress.

### 2.4. Exosome-Derived miRNA and PA

In human biological fluids, miRNAs accumulate within extracellular vesicles (EVs, including apoptotic bodies, microvesicles, and exosomes) and bind to macromolecules, such as the AGO2 protein or lipoproteins [56]. Exosomes play a key role in the cells’ cross-talk and in the pathogenesis of human diseases.

Recently, our group explored the function of miRNAs in IPAs and the therapeutic strategy of exosome-derived miRNAs for the disease [20]. Twenty DE miRNAs were identified; the two lowest miRNAs, miR-99a-3p and miR-149-5p, were used for the subsequent studies. We found that exosome-derived miR-99a-3p and miR-149-5p inhibited cell viability, migration, and tube formation [20]. Therefore, this study suggested that the upregulation of miR-99a-3p and miR-149-5p can inhibit the progression of IPA by the exosome, and exosome-derived miRNAs represent good potential candidates for future therapies for IPA.

Acromegaly, an endocrine and metabolic disease caused by GHPAs, is partially attributable to an excessive function of the GH and insulin-like growth factor-1 (IGF1) hormones [57,58,59]. In addition, GHPA-derived exosomes contain miRNAs and proteins that regulate cell proliferation and differentiation in distal extremities. Xiong et al. found that GHPA-derived exosomes may be involved in bone formation and osteoblast proliferation via promoting cell viability and DNA replication [37]. Furthermore, they found that exosome-derived miR-21-5p stimulated osteoblast information in the GH/IGF1 pathway. Taken together, exosome-derived miR-21-5p may be a candidate biomarker for the treatment of acromegaly.

RNA-seq was used for identifying exosome-derived miRNAs in six somatotroph adenomas and six healthy pituitary samples [60]. In this study, a total of 169 DE exosomal miRNAs were identified, and miR-423-5p was significantly downregulated in the somatotroph adenoma, whereas pituitary tumor transforming gene (PTTG1), a target of miR-423-5p, was upregulated in patients and contributed to the promotion of proliferation and migration of somatotropic adenoma cells. Thus, their findings indicate that exosome-derived miRNAs, especially miR-423-5p, may be valuable biomarkers for the development of new therapeutic strategies. Next-generation sequencing showed that miR-26b-5p, miR-126-5p, miR-148b-3p, and miR-150-5p were detected in patients with FSH/LH+ adenoma plasma samples, and also in the exosomes of the patients, but others (i.e., miR-6514-3p, miR-6850-5p, and miR-6867-5p) were not detectable in plasma and exosomes [24]. Interestingly, the authors said that the decrease in miR-143-3p in the plasma may be a potential biomarker for patients with FSH/LH+ adenoma after transsphenoidal surgery, but the expression of exosome-derived miR-143-3p in FSH/LH+ samples was not significant, indicating that miR-143-3p is mainly expressed in protein-associated plasma rather than in exosomes.

In conclusion, exosomes are effective markers and promising new therapeutic targets for PA and its complications. However, the role of exosomes, especially intercellular communication, in the pathogenesis of PA needs further investigation.

## 3. lncRNA and PA

Long non-coding RNAs (lncRNAs) are non-coding RNAs with over 200 nucleotides that cannot encode peptides [61,62]. A variety of functions of lncRNAs have been discovered, such as regulating gene activation and silencing [63,64], post-translational regulation [65,66,67], alternative splicing [68], and X chromosome modification [69]. LncRNAs perform these functions through different mechanisms, including acting as competing endogenous RNAs (ceRNAs) that sponge miRNAs or proteins [70], promoting or inhibiting long-range chromatin interactions [71,72], acting as molecular scaffolds for guiding chromatin-modifying enzymes [73,74,75], and even functioning through the behavior of transcription itself [64,76]. Given the important roles of lncRNAs in the regulation of epigenetic processes, the dysregulation of lncRNAs is implicated in many human diseases [9,77,78,79]. In addition, increasing studies have shown that lncRNA is involved in the progression, proliferation, apoptosis, autophagy, and metastasis of PA. In this section, available data on the role of dysregulated lncRNAs in the pathogenesis of PA will be discussed. Table 2 summarizes DE lncRNAs in PA and its subtypes.

It is well known that miRNAs are processed from primary miRNA transcripts, and some lncRNAs can serve as host transcripts of miRNAs [77,80,81,82]. MIR205HG is a lncRNA that harbors the gene of miR-205. Du et al. showed that MIR205HG regulated growth hormone levels in the anterior pituitary gland independent of miR-205 and regulated the expression of growth hormone and prolactin by interacting with the transcription factor Pit1 [80]. Emerging technologies of high-throughput sequencing and microarray analysis have contributed to systematically identifying abundant lncRNAs. In 2017, a genome-wide study found that there were 839 DE lncRNAs between PA and normal tissues, and some of them might be related to the mTOR signaling pathway [83]. In a microarray analysis, a total of 113 DE lncRNAs were identified in NFPA [84]. The most significantly DE lncRNA, n334366, is a unique satellite in the co-expression network, where it is related to the neuroactive ligand–receptor interaction pathways [84]. Subsequently, a total of 246 DE lncRNAs were identified between IPA and NIPA using a lncRNA microarray analysis [85]. The LOC105371531, LOC105375785, and FAM182B expression levels were significantly decreased in IPA compared to NIPA according to qRT-PCR [85]. ROC curve analysis showed that the expression of LOC105375785 and FAM182B could distinguish IPA from NIPA, suggesting that LOC105375785 and FAM182B might be implicated in the invasiveness of IPAs and might be novel biomarkers for the diagnosis of IPAs. Subsequently, the transcriptional expression of 684 DE lncRNAs was compared between bone invasive pituitary adenoma (BIPA) and NIPA [86]. The inflammatory factor TNF-α, regulated by the lncRNA SNHG24, plays an important role in BIPA [86], suggesting that they might be therapeutic targets.

Moreover, some common lncRNAs have also been investigated in patients with PA. H19 is the first encoding lncRNA discovered and plays opposite roles in different tumors [87,88,89]. The expression of H19 was significantly upregulated in invasive GHPA compared to that in noninvasive GHPA, suggesting that H19 might be a diagnostic and therapeutic target for GHPA [90]. Since H19 knockdown contributes to inhibiting the activation of the NF-κB pathway, it has been speculated that H19 may increase the invasiveness of PA by affecting the transcription of target genes via the NF-κB pathway [91]. Additionally, H19 expression was downregulated in human PA tissues and was negatively correlated with disease progression, while the upregulation of H19 inhibited tumor growth and cell proliferation [92]. Mechanistically, H19 could block mTORC1-mediated 4E-BP1 phosphorylation. These findings indicate that H19 may be a potential target for PA. Notably, exosome-derived H19 was also significantly downregulated in PA tissues and GH3 cells [93]. Exosome-derived H19 expression was negatively correlated with the progression of PA and used as a prognostic biomarker for patients treated with prolactinomas. Mechanistic studies found that H19 inhibited the growth of distal pituitary tumors by regulating 4E-BP1 phosphorylation [93]. Therefore, plasma exosome-derived H19 may be a significant target for predicting prolactinomas’ responses.

In conclusion, lncRNAs are promising therapeutic targets, and the development of these therapies requires the identification of some cell- or tissue-specific lncRNAs, but it remains challenging. In addition, exosome-derived lncRNAs and the cross-talk between lncRNAs and miRNAs are also promising research directions.

**Table 2:** Dysregulation of lncRNA in different PA types.

Author (Ref.)	Type of PA	Sample	lncRNA	Expression	Target Gene	Biological Function
Peng [85]	IPA	Tissues	FAM182B, LOC105371531, LOC105375785	Down	NA	FAM182B and LOC105375785 can distinguish IPA from NIPA.
Cheng [94]	NFPA	Tissues	LOC101927765, RP11-23N2.4, RP4-533D7.4	NA	NA	High prediction accuracy for NFPA recurrence.
Lu [95]	PA	Tissues, HP75 cell lines	IFNG-AS1	Up	ESRP2	An oncogene that promoted tumor progression.
Lu [90]	GHPA	Tissues	H19, MALAT-1	Up	NA	NA
Fu [96]	PA	Tissues, HP75 cells	CCTA2	Up	PTTG1	Poor prognosis, cell proliferation, migration, and invasion.
Li [97]	NFPA	Tissues	MEG, HOTAIR	MEG3: Down; HOTAIR: Up	PCNA	Tumor development and invasion.
Cheng [98]	NFPA	Tissues	COA6-AS1, RP11-23N2.4	Up	NA	Tumor regrowth with a high predictive accuracy.
Zhang [99]	PA	Tissues, GH3 and HP75 cells	PVT1	Up	NA	Cell migration, proliferation, and EMT.
Zhang [93]	GHPA, NFPA, PRLPA	Exosome, GH3 cells	H19	Down	4E-BP1	The prognosis or drug response.
Xing [84]	NFPA	Tissues	n334366, n335657, n409198, MEG3, n337303, n340496, n334406, n332607, n333074, n332409	Down: n334366, n335657, n409198, MEG3. Up: n337303, n340496, n334406, n332607, n333074, n332409	NA	Tumorigenesis.

Ref., reference; NA, not available; EMT, epithelial–mesenchymal transition.

## 4. circRNA and PA

Most circular RNA (circRNA), a single-stranded, endogenous non-coding RNA, is produced from the back-splicing of exons of precursor mRNAs and is abundant and highly conserved in blood and disease [100,101]. Although circRNA has long been thought to be a transcriptional error, recent advances in RNA-seq and bioinformatics have shown that it plays an important role in human health and diseases [102]. The abnormal expression of circRNA is involved in various physiological and pathological processes [100,103,104], such as epithelial–mesenchymal transition, differentiation, metastasis, and tumorigenesis [105,106]. Several studies revealed that the dysregulation of circRNA is associated with many different tumors, including PA [107,108]. In this section, available data on DE circRNA in PA will be discussed. Table 3 summarizes DE circRNAs in PA and its subtypes.

The circRNA expression profile was identified between NFPA and PA, and the expression of circVPS13C was significantly increased in NFPA tissues and cells but was downregulated in patients’ serum 7 days after transsphenoidal adenoma resection. Mechanistically, the knockout of circVPS13C increased the expression of IFITM1 and activated MAPK/apoptosis-associated downstream genes; further studies showed that circVPS13C inhibited the expression of IFITM1 by competitively interacting with RRBP1. Therefore, circVPS13C is a critical regulator for the proliferation and development of NFPAs through a novel mechanism that regulates mRNA stability by interacting with ribosome-binding proteins [101]. Moreover, the circRNA signature has clinical application value in predicting recurrence and progression in NFPA. Two circRNAs (i.e., hsa_circ_0000066 and hsa_circ_0069707) were related to the progression-free survival in NFPA, and the two circRNAs had a high prediction accuracy for tumor recurrence [109].

In addition to the important role of circRNA in NFPA, it is also involved in GHPA. A circRNA microarray identified the DE circRNA profile in GHPA, of which 1938 circRNAs were upregulated and 1601 were downregulated [110]. Among all the DE circRNAs, hsa_circ_0001368 was significantly upregulated in GHPA and correlated with the invasiveness and serum GH level of GHPA. Further studies found that the knockdown of hsa_circ_0001368 significantly inhibited the cells’ proliferation, invasion, and serum GH level. Moreover, the expression of hsa_circ_0001368 was positively correlated with the pituitary-specific transcription factor Pit-1 [110]. Therefore, hsa_circ_0001368 may represent a novel therapeutic biomarker for GHPA.

In conclusion, although circRNAs have an important role in PA, studies on the association of circRNA with PA are currently limited. Further studies are highly encouraged to explore the role of circRNAs (especially exosome-derived circRNAs) and their downstream target genes or signaling pathways in the pathogenesis of PA.

**Table 3:** Dysregulation of circRNA in different PA types.

Author (Ref.)	Type of PA	Sample	circRNA	Expression	Target Gene	Biological Function
Hu [111]	NFPA	Tissues	hsa_circRNA_102597	Down	NA	Associated with tumor diameter and Knosp grade, differentiated invasive from noninvasive NFPAs, and predicted tumor progression and recurrence.
Zhang [101]	NFPA	Tissues, PDFS cells	CircVPS13C	Up	RRBP1	Promoted proliferation and development.
Du [110]	GHPA	Tissues	hsa_circ_0001368	Up	Pit-1	Associated with the invasiveness and serum GH level; promoted cell proliferation and invasion.
Guo [109]	NFPA	Tissues	hsa_circ_0000066 and hsa_circ_0069707	Up	NA	Associated with the PFS, and had a high prediction accuracy for tumor recurrence.
Wang [112]	NFPA	Tissues	hsa_circ_0054722, hsa_circ_0007362, hsa_circ_0012346, hsa_circ_0062222, hsa_circ_0016403, hsa_circ_0033349	Up: hsa_circ_0054722, hsa_circ_0007362, hsa_circ_0012346, Down: hsa_circ_0062222, hsa_circ_0016403, hsa_circ_0033349	NA	Contributed to diagnosis, prognosis, and clinical treatment.

Ref., reference; NA, not available; GH, growth hormone; PFS, progression-free survival.

## 5. ceRNA and PA

The competing endogenous RNA (ceRNA) hypothesis is that transcripts such as lncRNA and circRNA competitively bind to miRNAs through the miRNA response element (MRE), thus forming a complex regulatory network to realize their respective regulatory functions [113]. In this section, we discuss the available evidence for DE lncRNA and circRNA in PA, respectively. Table 4 summarizes the interactions between lncRNA/circRNA and miRNA in PA and its subtypes.

First, several upregulations of lncRNAs have been implicated in PA [114,115,116]. For example, lncRNA TUG1 was significantly downregulated in PA tissues and PA-associated cell lines [114]. The expression of TUG1 was associated with invasion, Knosp grade, and tumor size, and TUG1 silencing downregulated the expression of NF-κB p65 and κB (IκB)-α and TESC by targeting miR-187-3p [114], suggesting that TUG1 modulates PA progression by regulating the TESC–NF-κB signaling pathway via sponging miR-187-3p. However, lncRNA MEG3 was downregulated in PA tissues and PA-associated cells [117]. The overexpression of MEG3 inhibited the proliferation, invasion, migration, and epithelial–mesenchymal transition (EMT) process and accelerated PA-associated cells’ apoptosis. Further studies found that MEG3 negatively regulated miR-23b-3p expression, while miR-23b-3p negatively regulated FOXO4 expression [117]. Similar to the findings for MEG3 in PA, MEG3 was also decreased in NFPA [118]. Moreover, MEG3 is an enhancer of miR-376B-3P; the overexpression of MEG3 and miR-376B-3p inhibited tumorigenesis and promoted apoptosis; HMGA2, a target gene of miR-376B-3p, is an oncogene in PA and could be negatively regulated by MEG3 via enriching miR-376B-3p [118]. Therefore, the novel regulatory network of the MEG3/MIR-376B-3P/HMGA2 interactions in NFPAs may be helpful for anticancer treatments. In recent years, several studies have shown that some DE lncRNAs were upregulated in IPA tissues and cells; these lncRNAs participated in viability, migration, invasion, and epithelial-mesenchymal transition (EMT) by sponging miRNAs [119,120,121,122]. Therefore, these lncRNAs may be novel valuable therapeutic targets for IPA.

CircRNAs are primarily known to act as miRNA sponges or ceRNAs to regulate transcriptional activity [113]. CircOMA1 (hsa_circRNA_0002316) was significantly decreased but miR-145-5p was upregulated in NFPA samples [123]. The overexpression of miR-145-5p inhibited NFPA cell invasiveness and proliferation as well as promoting cell apoptosis, whereas circOMA1 can reverse these effects by sponging miR-125-5p. Therefore, circOMA1 promoted the tumorigenesis of NFPAs by acting as a sponge of the antioncogene miR-145-5p (123). Moreover, circNFIX (hsa-circ_0005660) was significantly upregulated but miR-34a-5p was downregulated in invasive PA tissues (124). The knockdown of circNFIX or overexpression of miR-34a-5p contributed to inhibiting cell proliferation, migration, and invasion, and circNFIX reversed the promoting effect of miR-34a-5p on the progression of PA by sponging miR-34a-5p (124). Therefore, circNFIX may be a promising target for the treatment of PA.

In conclusion, the interactions between miRNA and lncRNA/circRNA, especially the ceRNA mechanism, play an important role in the pathogenesis of PA and its subtypes.

**Table 4:** Interaction between lncRNA/circRNA and miRNA in different PA types.

Author (Ref.)	Type of PA	Sample	LncRNA/ circRNA	Expression	Target miRNA	miRNA Target	Biological Function
Du [123]	NFPA	Tissues, pituitary tumor-derived folliculostellate (PDFS) cell line	circOMA1	Down	miR-145-5p	TPT1	Promoted cell proliferation and invasiveness.
Cheng [124]	IPA	Tissues; GT1-1 and GH3 cells	circNFIX	Up	miR-34a-5p	CCNB1	Promoted cell invasion, migration, and proliferation.
Zhu [79]	BIPA	Tissues	MEG8	Up	miR-454-3p	TNF-α	Promoted bone destruction and associated with poor PFS.
Zhao [125]	IPA	Tissues; RC-4B/C and GH3 cells	PCAT6	Up	miR-139-3p	BRD4	Regulated the progression of PA.
Wu [78]	PRLPA	Tissues, GH3 cells	H19	Down	miR-93a	ATG7	H19 had a synergistic effect with dopamine agonist treatment on prolactinomas.
Yue [126]	PA	Tissues; GH1, RC-4B/C, GH3 and MMQ cell lines	SNHG7	Up	miR-449a	NA	Associated with poor survival outcomes; increased cell viability, migration, and invasion and decreased apoptosis.
Zhu [86]	BIPA	Tissues; GH3 and RAW264.7 cells	NR_033258, SNHG24	Up	miR-181c-5p, miR-454-3p	NA	TNFα induced osteoclast differentiation, and NR_033258 and SNHG24 regulated TNFα expression.
Qiu [119]	PA	HP75 cells	LINC01004	Up	miR-323a-3p/miR-136-5p	RCN2	Promoted disease progression.
Li [120]	IPA	GH3 and HP75 cells	KCNQ1OT1	Up	miR-140-5p	RAB11A	Promoted the EMT, cellular stemness, and proliferation and invasion.
Wu [115]	PA	Tissues; RC-4B/C, GH3, and MMQ cells	BBOX1-AS1	Up	miR-361-3p	E2F1	Promoted cell invasion, apoptosis, and proliferation.
Huang [116]	PA	HP75 cells	LINC01116	Up	miR-744-5p	HOXB8	Promoted cell proliferation, migration, and EMT process.
Li [121]	IPA	Tissues; AtT-20 and GT1-1 cells	LINC00473	Up	miR-502-3p	KMT5A	Promoted cell proliferation.
Zhang [114]	PA	Tissues; HP75 and GH3 cells	TUG1	Up	miR-187-3p	TESC	Promoted cell proliferation, invasion, migration, and EMT, and inhibited apoptosis.
Mao [122]	IPA	Tissues, HP75 cells	SNHG6	Up	miR-944	RAB11A	Promoted cell viability, migration, invasion, and EMT.
Wang [117]	PA	Tissues; GH3 and MMQ cells	MEG3	Down	miR-23b-3p	FOXO4	Promoted cell proliferation, apoptosis, and the EMT process.
Zhu [118]	NFPA	Tissues, PDFS cells	MEG3	Down	miR-376B-3p	HMGA2	Tumorigenesis and cell apoptosis

Ref., reference; NA, not available; EMT, epithelial–mesenchymal transition; PFS, progression-free survival.

## 6. Conclusions and Future Perspective

The abundance of DE ncRNAs in PA has been widely reported. Investigating the roles of ncRNAs is helpful for understanding the occurrence and development of PA, and some ncRNAs can be used as biomarkers for the detection of PA [1,18,43,127], while other ncRNAs can contribute to the diagnosis of PA [52,85,90,112], distinguish the disease subtypes [24,25,34,93], and predict tumor invasion and progression [20,26,35,85,95,110,111,123]; the remaining ncRNAs may be implicated in the pathogenesis of PA [116,117,120,122]. In addition, the regulatory networks of lncRNA/circRNA and miRNA provide promising biomarkers for the noninvasive diagnosis and molecular therapy of PA, and exosome-derived ncRNAs bring a tremendous infusion of hope to the improvement of the disease diagnosis and future potential therapeutic targets for treatments. Therefore, available data on the DE ncRNAs in PA were reviewed and discussed in this study.

**Figure 1:**
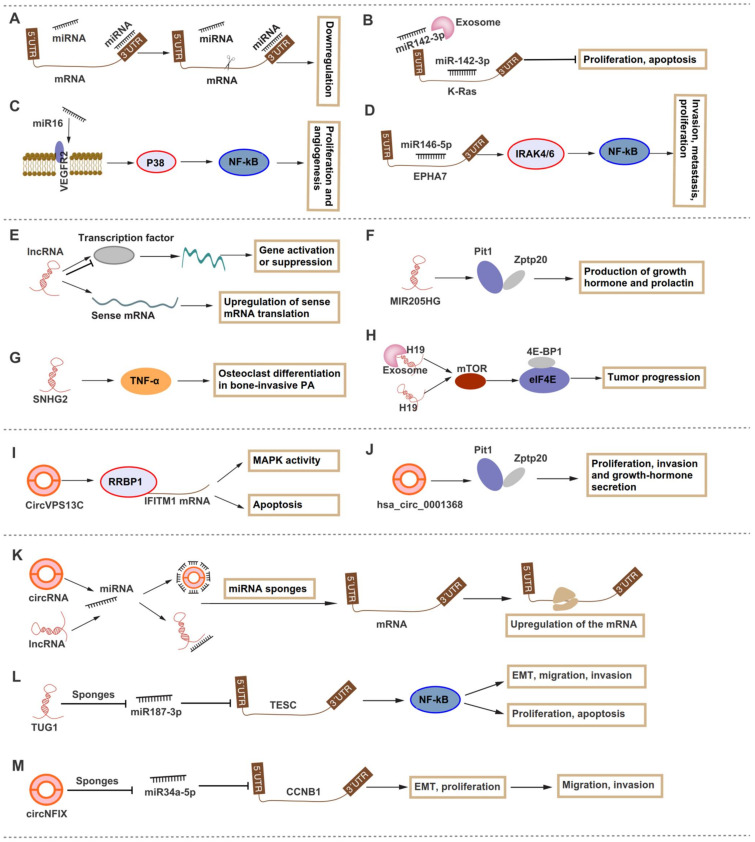
The potential mechanisms of non-coding RNAs in pituitary adenoma. (**A**) miRNA inhibits protein synthesis by binding to the 3’-UTR of mRNA. (**B**–**D**) miR-142-3p, miR-16, miR-146-5p, and exosome-derived miR-146-5p are involved in the pathogenesis of pituitary adenoma (PA). (**E**) LncRNA regulates gene activation or suppression by increasing or decreasing transcription via the transcription factor, respectively; antisense lncRNA upregulates the sense mRNA translation process. (**F**–**H**) MIR205GH, SNHG24, H19, and exosome-derived H19 are involved in the pathogenesis of PA. (**I**–**J**) CircVPS13C and hsa_circ_0001368 are involved in the pathogenesis of PA. (**K**) CircRNA and lncRNA increase the translation process and protein production via sponging miRNA. (**L**) LncRNA TUG1 modulates PA progression by regulating the TESC–NF-kB pathway via sponging miR-187-3p. (**M**) CircNFIX promotes PA progression by regulating CCNB1 via sponging miR-34a-5p.

Among the abundant DE ncRNAs, miRNAs are the most widely studied. We discuss the role of miRNAs in different PA subtypes in tissues, biofluids, and cell-free environments [17,18,19,20,37]. miRNAs inhibit the expression of target genes at the post-transcriptional level by pairing with the 3′-UTR of mRNA (Figure 1); thus, miRNAs are involved in various pathological and physiological processes by playing a negative role in cell proliferation, differentiation, metabolism, and apoptosis. PA rarely progresses to malignancy but usually presents as aggressive growth, and miRNAs are related to excessive cell proliferation and apoptosis, and tumor size. Therefore, upregulated miRNAs may inhibit the expression of antioncogenes, while downregulated miRNAs cannot inhibit the expression of oncogenes. Moreover, miRNAs play an important role in the diagnosis and prognosis of PA. However, developing biomarkers based on DE miRNAs is inaccurate, as they are also altered in other cancers [128]. To address this issue, here are some research directions for future studies. Firstly, specific miRNAs from specimens should be developed. PA-specific tissue-/serum- and extracellular vesicle (e.g., exosome)-derived miRNAs are highly recommended. Secondly, combined diagnosis should be conducted. Future studies could measure different ncRNAs (e.g., lncRNAs, circRNA, and miRNAs) simultaneously, and perform combined diagnosis to improve the accuracy. In addition, miRNAs can be combined with patients’ medical records, clinical parameters, and radiological examinations to reach an adequate level of accuracy. Importantly, exosome-derived miRNAs participated in cross-talk and transformation between different cells, which raises the possibility of miRNA as circulating biomarkers [129].

Studies on the association of lncRNAs and circRNAs with PA are limited compared to those for miRNAs. On the one hand, lncRNAs and circRNAs participate in PA by regulating the expression of target genes [95,96,101,110]. On the other hand, many studies have shown that the two ncRNAs regulate mRNA by sponging miRNAs [78,79,123,124]. The dysregulation of lncRNAs regulates transcription by controlling the function of transcription factors, resulting in the overexpression of oncogenes or underexpression of antioncogenes. Similar to the role of miRNA in PA, these DE lncRNAs and circRNAs have also been identified in cancers other than PA, suggesting that the development of PA-specific lncRNAs and circRNAs requires more in-depth mechanism studies. Cross-talk between lncRNAs/circRNAs and miRNA, especially ceRNAs, will help us to understand the role of lncRNAs and circRNAs in the pathogenesis of PA [117,121,123,124], and potential regulatory mechanisms of lncRNAs, circRNAs, and miRNAs in PA are shown in Figure 1. In addition, few studies have explored the involvement of exosome-derived lncRNAs and circRNAs in PA, excepting the relationship between exosomal H19 and PA [93]. Therefore, the function of exosome-derived ncRNAs in the pathogenesis of PA remains to be investigated, especially the cross-talk of ncRNAs in different cells mediated by exosomes.

Although increasing studies have been performed to explore the relationship between ncRNAs and PA, the exact mechanism of PA caused by ncRNAs is still unclear. Therefore, some suggestions are highly recommended. Firstly, well-designed experiments should involve a particular signaling pathway to identify specific ncRNAs that are closely associated with PA. Secondly, unknown targets of ncRNAs, including miRNAs targeted by lncRNAs and circRNAs, and mRNAs targeted by the three ncRNAs, should be identified. Thirdly, the pathogenesis of PA and its subtypes should be investigated in combination with population studies and basic experiments as well as combination with genetics and epigenetics. Finally, it is urgent to develop novel drugs effective against ncRNA-associated targets and conduct related clinical trials for the treatment of PA.

With a better understanding of the molecular mechanisms and signaling pathways of cell- and tissue-specific ncRNAs in PA, it is believed that novel clinical diagnostic markers and targeted treatments for PA and its subtypes may be implemented.

## Data Availability

Not applicable.
